# Using a multi-staged strategy based on machine learning and mathematical modeling to predict genotype-phenotype risk patterns in diabetic kidney disease: a prospective case–control cohort analysis

**DOI:** 10.1186/1471-2369-14-162

**Published:** 2013-07-23

**Authors:** Ross KK Leung, Ying Wang, Ronald CW Ma, Andrea OY Luk, Vincent Lam, Maggie Ng, Wing Yee So, Stephen KW Tsui, Juliana CN Chan

**Affiliations:** 1 Hong Kong Bioinformatics Centre, The Chinese University of Hong Kong, Hong Kong, SAR, China; 2 Department of Medicine and Therapeutics, The Chinese University of Hong Kong, Hong Kong, SAR, China; 3 Li Ka Shing Institute of Health, Hong Kong, China; 4 School of Biomedical Sciences, Hong Kong, China; 5 Hong Kong Institute of Diabetes and Obesity, The Chinese University of Hong Kong, The Prince of Wales Hospital, Shatin New Territories, Hong Kong SAR, China

**Keywords:** Prediction, Diabetic kidney disease, Genotypes, Phenotypes, Machine learning, Random forest, Support vector machine

## Abstract

**Background:**

Multi-causality and heterogeneity of phenotypes and genotypes characterize complex diseases. In a database with comprehensive collection of phenotypes and genotypes, we compared the performance of common machine learning methods to generate mathematical models to predict diabetic kidney disease (DKD).

**Methods:**

In a prospective cohort of type 2 diabetic patients, we selected 119 subjects with DKD and 554 without DKD at enrolment and after a median follow-up period of 7.8 years for model training, testing and validation using seven machine learning methods (partial least square regression, the classification and regression tree, the C5.0 decision tree, random forest, naïve Bayes classification, neural network and support vector machine). We used 17 clinical attributes and 70 single nucleotide polymorphisms (SNPs) of 54 candidate genes to build different models. The top attributes selected by the best-performing models were then used to build models with performance comparable to those using the entire dataset.

**Results:**

Age, age of diagnosis, systolic blood pressure and genetic polymorphisms of uteroglobin and lipid metabolism were selected by most methods. Models generated by support vector machine (svmRadial) and random forest (cforest) had the best prediction accuracy whereas models derived from naïve Bayes classifier and partial least squares regression had the least optimal performance. Using 10 clinical attributes (systolic and diastolic blood pressure, age, age of diagnosis, triglyceride, white blood cell count, total cholesterol, waist to hip ratio, LDL cholesterol, and alcohol intake) and 5 genetic attributes (*UGB G38A*, *LIPC* -*514C* > *T*, *APOB Thr71Ile*, *APOC3 3206T* > *G* and *APOC3 1100C* > *T*), selected most often by SVM and cforest, we were able to build high-performance models.

**Conclusions:**

Amongst different machine learning methods, svmRadial and cforest had the best performance. Genetic polymorphisms related to inflammation and lipid metabolism warrant further investigation for their associations with DKD.

## Background

The prevalence of diabetic kidney disease (DKD) is rising in parallel to the growing epidemic of type 2 diabetes and obesity in both developing and industrialized societies [1]. The development of DKD is due to complex interactions between multiple modifiable risk factors such as hypertension, hyperglycaemia and dyslipidaemia and genetic variants [2]. Recent genome wide association studies (GWAS) have uncovered novel loci for complex traits such as type 1 diabetic nephropathy with odds ratio of 1.1-1.2 [3]. While there are ongoing efforts to discover genomic structural and regulatory variations to explain the heritability of these complex traits [4], other researchers argued that for common diseases due to common variants, as few as 20 loci may explain 50% of the population attributable risk. The challenge lies in unraveling the nature of these gene-gene interactions and their impacts on phenotypes and clinical outcomes [5].

In this post-GWAS era, in addition to conventional statistical methods such as chi-square test or logistic regression, machine learning methods are other tools which can be used to identify novel relationships between genetic variations and disease susceptibility [6-8]. These computational applications enable researchers to uncover hidden patterns, reclassify data and present their inter-relationships in an understandable way for decision making. However, the applicability and utility of these computational tools in common diseases such as type 2 diabetes have not been fully explored and utilized. We applied seven machine learning methods to a comprehensive database with detailed phenotypes and genotypes of candidate genes for vasculopathy to identify optimal combinations of clinical and/or genetic factors predictive of DKD. These seven machine learning methods included two decision trees (the classification and regression tree and the C5.0 decision tree), random forest, naïve Bayes classifier, neural network, partial least squares regression and support vector machine.

## Methods

### Subjects

Between 1^st^ July 1994 and 30^th^ June of 1998, a consecutive cohort of 1,386 Chinese patients with type 2 diabetes were enrolled into the Hong Kong Diabetes Registry with documentation of risk factors, complications, drug use and clinical outcomes. None of the patients had history of unprovoked ketosis and did not require insulin within the first year of diagnosis. The study protocol was approved by the Clinical Research Ethics Committee of the Chinese University of Hong Kong. All patients gave written informed consent and donated their clinical data and DNA for research and publication purposes. Declaration of Helsinki was adhered to in the study.

### Clinical assessment and laboratory assay

All patients underwent a structured 4-hour clinical and biochemical assessment, details of which have been described [9]. In brief, anthropometric measurements and blood pressure (BP) were obtained. Drug use and past medical history pertaining to vascular diseases were documented. Physical assessment for retinopathy, sensory neuropathy and peripheral arterial disease was performed using standard methodologies. Fasting blood samples for plasma glucose, glycated hemoglobin, lipid profile and renal function, as well as a random spot urine sample for albumin to creatinine ratio (ACR) were collected. Estimated glomerular filtration rate (eGFR) was calculated using the Chinese-modified Modification of Diet in Renal Disease equation [10]. Diabetic kidney disease was defined as eGFR less than 60 ml/min/1.73 m^2^[11]. All patients were censored on 30^th^ July 2005. Data were retrieved from the Hospital Authority Central Computer System, using the Hong Kong Identity Card number, which is compulsory for all residents in Hong Kong.

### Genotyping

Genotyping was performed using line arrays from Roche Molecular Systems comprising 107 single nucleotide polymorphisms (SNPs) in 65 candidate genes related to lipid and homocysteine metabolism, inflammation, thrombosis, endothelial function, stress and natriuretic responses. The selection of these genes was based on published studies on their biological plausibility and risk associations with cardiovascular diseases, immune response and inflammation [12,13] (http://www.ncbi.nlm.nih.gov/gene?term=gene, accessed 1 June 2007). In addition, we genotyped the *ALR2* (aldose reductase) polymorphisms based on known association between this genetic variant and DKD. The method for genotyping of the *ALR2* polymorphism has been described [14]. Genotype call rate, Hardy-Weinberg equilibrium and minor allele frequency for each SNP was assessed using PLINK (V.0.99, http://pngu.mgh.harvard.edu/~purcell/plink/download.shtml) in the study population. After excluding SNPs with call rate less than 95%, P value < 0.05 for Hardy-Weinberg equilibrium and/or minor allele frequency < 0.01, 79 SNPs of 55 genes were included in the present analysis. Full details of these SNPs are available in Additional file 1.

### Patient selection

From the cohort of 1,386 type 2 diabetic patients, we excluded 500 patients due to missing eGFR at baseline or end of follow-up. Those who had normal renal function at baseline but progressed to develop DKD (n = 80) and those who had DKD at baseline but then regressed to have normal renal function (n = 6) were excluded. To reduce confounding effects due to patients with inconclusive renal status, we only included patients with consistent eGFR at baseline and end of follow-up, i.e. less than 55 ml/min/1.73 m^2^ for DKD (n = 119) or more than 65 ml/min/1.73 m^2^ for non-DKD (n = 554).

### Selection of variables

We removed parameters indicative of renal function to discover novel predictors. These included urinary ACR and serum creatinine at baseline. We also excluded drug data due to confounding effects of drug indications, i.e. patients with more risk factors were more likely to need treatment. For variables with close inter-correlations, we only selected one of them for analysis. Finally, we excluded variables with zero- or near zero-variance, leaving 87 (17 clinical and 70 SNPs of 54 candidate genes) attributes for model development. These attributes were then grouped into three categories for input into various machine learning programs: 1) clinical and genetic attributes; 2) genetic attributes only; and 3) clinical attributes only.

### Imputation of missing values and handling of imbalanced data

We imputed the missing values by exploring similarities between cases. Firstly, we identified the 10 most similar cases and calculated the Euclidean distance between the values of cases and used the median value to impute the missing value. To adjust for class imbalance, we applied the Synthetic Minority Over-sampling Technique, which generated new examples of the minority class (those with DKD) using the nearest neighbors of these cases and under-sampled the majority class examples (those without DKD) [15].

### Statistical analysis

All statistical analyses were performed using the SPSS Statistics 17.0 (SPSS Inc. Chicago) unless otherwise specified. The clinical data were expressed as median (inter-quartile range, IQR) or percentages. The Mann–Whitney Two-Sample test and Chi-square test were used as appropriate. A P value less than 0.05 (2-tailed) was considered significant.

### Model training and parameter tuning

We applied and compared the following machine learning methods: partial least square regression, the classification and regression tree, the C5.0 decision tree, random forest, naïve Bayes classification, neural network and support vector machine. All the machine learning methods were performed under the R computing environment. The details of package versions and parameters used for each machine learning method were described in Additional file 2.

Seventy-five percent of the data were partitioned into the training set and the remaining, into the testing set. For each machine learning method, ten sets of parameters were tested. For each set of parameters, 10-fold cross validation was performed to obtain an average value of the performance across hold-out predictions. Receiver operator curve (ROC) analysis was used to select the optimal model using the largest value of area under the curve (AUC). We then estimated the performance of the trained models by subjecting them to the testing dataset to predict DKD. The machine learning methods with the best performance were then used in a second stage to select a subset of important variables to develop models with performance comparable to that using the entire dataset. In ranking the importance of variable, we adopted the conditional importance in random forest [16] and Variance Importance in Projection in Partial Least Squares [17], to avoid bias generated due to use of predictor variables with different scales and variable collinearity in our dataset. For the other machine learning methods, the default variable importance specified in the caret package (5.15-0.52) was adopted.

## Results

In this prospective cohort of 673 patients with type 2 diabetes, 41.2% were male, the median age was 57 (IQR: 48 to 65) years and the median duration of diabetes was 9 (IQR 3 to 13) years. Compared to patients without DKD, patients who had DKD were older and more obese. They also had higher BP, urinary ACR and worse lipid profile and were more likely to be treated with angiotensin converting enzyme inhibitors (ACEI) or angiotensin receptor blockers (ARB), anti-hypertensive drugs, lipid lowering drugs and insulin at baseline (Table 1). After a median follow-up period of 7.8 (IQR: 5.2 to 9.2) years, median eGFR was 38.0 (IQR: 27.0 to 48.9) and 119.9 (IQR: 101.7 to 138.1) ml/min/ 1.73 m^2^ for patients with DKD and those without DKD, respectively. The Additional file 3 compares the data at baseline and end of follow up between patients included and excluded from the analysis due to incomplete phenotypes. The excluded patients were older, had shorter duration of diabetes, higher LDL cholesterol, total cholesterol (TC), urinary ACR and lower eGFR than included patients.

**Table 1:** Baseline characteristics of 673 Chinese patients with type 2 diabetes stratified by the onset of diabetic kidney disease (DKD) after a median follow up period of 8 years

	**Subjects without DKD at baseline and 8 year**	**Subjects with DKD at baseline and 8**-**year**	**P value**
Number	554	119	
**Clinical features**			
Age (years)	56	64	<0.001^b^
	(47 to 63)	(58 to 69)	
Male sex	39.7% (220)	47.9% (57)	0.100^a^
Age of onset (years)	45	53	<0.001^b^
	(38–55)	(45–60)	
Duration of diabetes (years)	9	10	0.032^b^
	(2 to 13)	(6 to 13)	
Smoking			0.003^a^
Ex smokers	31.8% (176)	20.2% (24)	
Current smokers	9.7% (54)	20.2% (24)	
BMI (kg/m^2^)	24.7	25.2	0.160^b^
	(22.3 to 27.0)	(22.7 to 27.2)	
Waist circumference (cm) Men	88.0	89.0	0.325^b^
	(83.0 to 92.8)	(84.0 to 96.0)	
Waist circumference (cm) Women	83.0	85.0	0.109^b^
	(77.0 to 89.0)	(77.8 to 93.0)	
Waist to hip ratio	0.88	0.91	<0.001^b^
	(0.84 to 0.92)	(0.87 to 0.96)	
Systolic BP (mmHg)	132	156	<0.001^b^
	(120 to 145)	(140 to 171)	
Diastolic BP (mmHg)	77	83	<0.001^b^
	(70 to 85)	(76 to 93)	
**Laboratory data**			
HbA1c (%)	7.5	7.6	0.919^b^
	(6.7 to 8.7)	(6.6 to 8.8)	
Fasting plasma glucose (mmol/L)	7.9	8.3	0.849^b^
	(6.5 to 10.5)	(6.2 to 10.1)	
LDL cholesterol (mmol/L)	3.20	3.70	0.008^b^
	(2.70 to 3.90)	(2.80 to 4.38)	
HDL cholesterol (mmol/L)	1.20	1.11	<0.001^b^
	(1.00 to 1.50)	(0.90 to 1.40)	
Triglyceride (mmol/L)	1.25	1.87	<0.001^b^
	(0.87 to 1.95)	(1.14 to 2.55)	
Total cholesterol (mmol/L)	5.3	5.7	0.001^b^
	(4.6 to 6.0)	(4.8 to 6.7)	
White blood cell count (×10^9^/L)	7.0	7.7	<0.001^b^
	(5.8 to 8.3)	(6.8 to 9.1)	
ACR (mg/mmol)	1.5	245.6	<0.001^b^
	(0.8 to 4.7)	(81.5 to 423.4)	
eGFR (ml/min/ 1.73 m^2^)	119.9	38.0	<0.001^b^
	(101.7 to 138.1)	(27.0 to 48.9)	
**Drug use at baseline**			
Lipid lowering drugs	5.4% (30)	23.5% (28)	<0.001^a^
ACEI/ARB	6.1% (34)	28.6% (34)	<0.001^a^
Other blood pressure lowering drugs	21.3% (118)	63.9% (76)	<0.001^a^
Oral blood glucose lowering drugs	53.2% (295)	36.1% (43)	0.001^a^
Insulin	16.8% (93)	39.5% (47)	<0.001^a^

^a^ Derived from Chi-square text, % (N);^b^ Mann–Whitney Two-Sample Test, Median (25th to 75th quartiles).

Abbreviation: *BMI*, body mass index; *HbA1c*, glycated hemoglobin; *HDL*, high density lipoprotein; *LDL*, low density lipoprotein.

**Figure 1:**
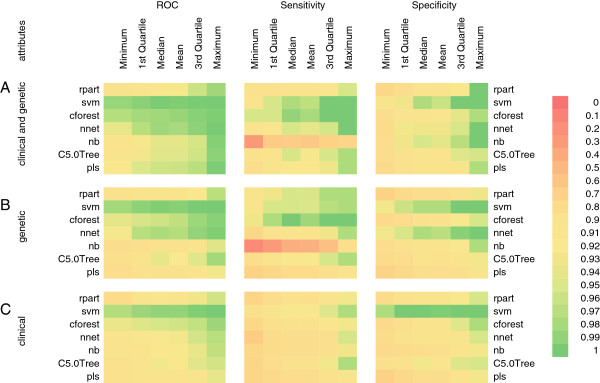
**Ten-fold cross-validation predictive performance by different machine learning methods in the DKD training dataset using A) clinical and genetic attributes, B) genetic-only attributes, C) clinical-only attributes.** Abbreviations – svmradial: support vector machine using radial basis kernel function, rpart: recursive partitioning and regression trees, nnet: feed-forward neural networks and multinomial log-linear models, nb: naïve Bayes classifier, cforest: random forest utilizing conditional inference trees as base learners, C5.0 Tree: C5.0 decision tree, pls: partial least squares regression.

If we included urinary ACR and serum creatinine in the model building process, all machine learning methods predicted correctly (i.e. accuracy = 1). All models had comparable performance if drug treatments were included in the dataset. Therefore, we excluded these attributes to identify subjects with hidden patterns for early intervention. We compared the performance of different machine learning methods using different sub-groups of attributes: 1) clinical and genetic; 2) genetic only; 3) clinical only (Figure 1). Naïve Bayes classification (nb) and partial least squares regression (pls) had the least optimal performance in the training stage. In general, support vector machine (svmRadial) and random forest (cforest), followed by Neural network (nnet) had the best performance for all sub-groups of attributes. svmRadial was least sensitive to parameter tuning and maintained good performance during data cross-validation.

**Figure 2:**
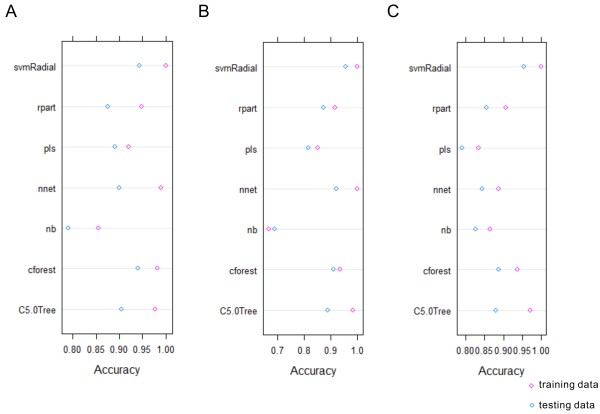
**Prediction accuracy by different machine learning methods in the DKD training and testing datasets using A) clinical and gene attributes, B) genetic-only attributes, C) clinical-only attributes.** Circles in pink and blue represent prediction accuracy using training and testing data respectively.

When applying the best fit models to the testing data, svmRadial and cforest outperformed the other machine learning methods (Figure 2). If we only used genetic attributes, nnet slightly performed better than cforest. Some machine learning methods were more selective in their input variables, e.g. pls had better performance when a larger number of attributes were available for model building and prediction. While nnet could not achieve comparable performance without genetic attributes, nb could not do so without clinical attributes.

Except for C5.0 Tree, clinical factors were preferentially selected for model building (Additional file 4, Figure A) and accounted for most of the variance of DKD. In particular, systolic BP and age were the two most important variables in models using clinical factors alone or in combination with genetic factors. For the genetic variants, polymorphisms of genes encoding uteroglobin (*UGB G38A*), hepatic lipase (*LIPC* -*514C* > *T*) and apolipoprotein B (*APOB Thr71Ile*) were most preferentially selected for model building (Additional file 4).

Since svmRadial and cforest had the best prediction performance, we were interested in identifying a smaller set of clinical and/or genetic attributes to build models with performance similar to that using all 87 attributes. To avoid bias from a single machine learning method, we first extracted the top 20 ranking attributes in all three sub-groups of “genetic”, “clinical” and “both” factors from the svmRadial and cforest models. We then selected the variables that appeared in the lists of “genetic” and “both” as well as that of “clinical” and “both” for each models. Finally, we selected variables which appeared in both the svmRadial and cforest models which yielded 15 attributes in total. To give an example, in the cforest model, TC was ranked the 7^th^ in the genetic + clinical model as well as the clinical model. In the svmRadial model, TC was ranked the 6^th^ in the genetic + clinical model and the 9^th^ in the clinical model and thus TC was used to build the most optimal model.

**Figure 3:**
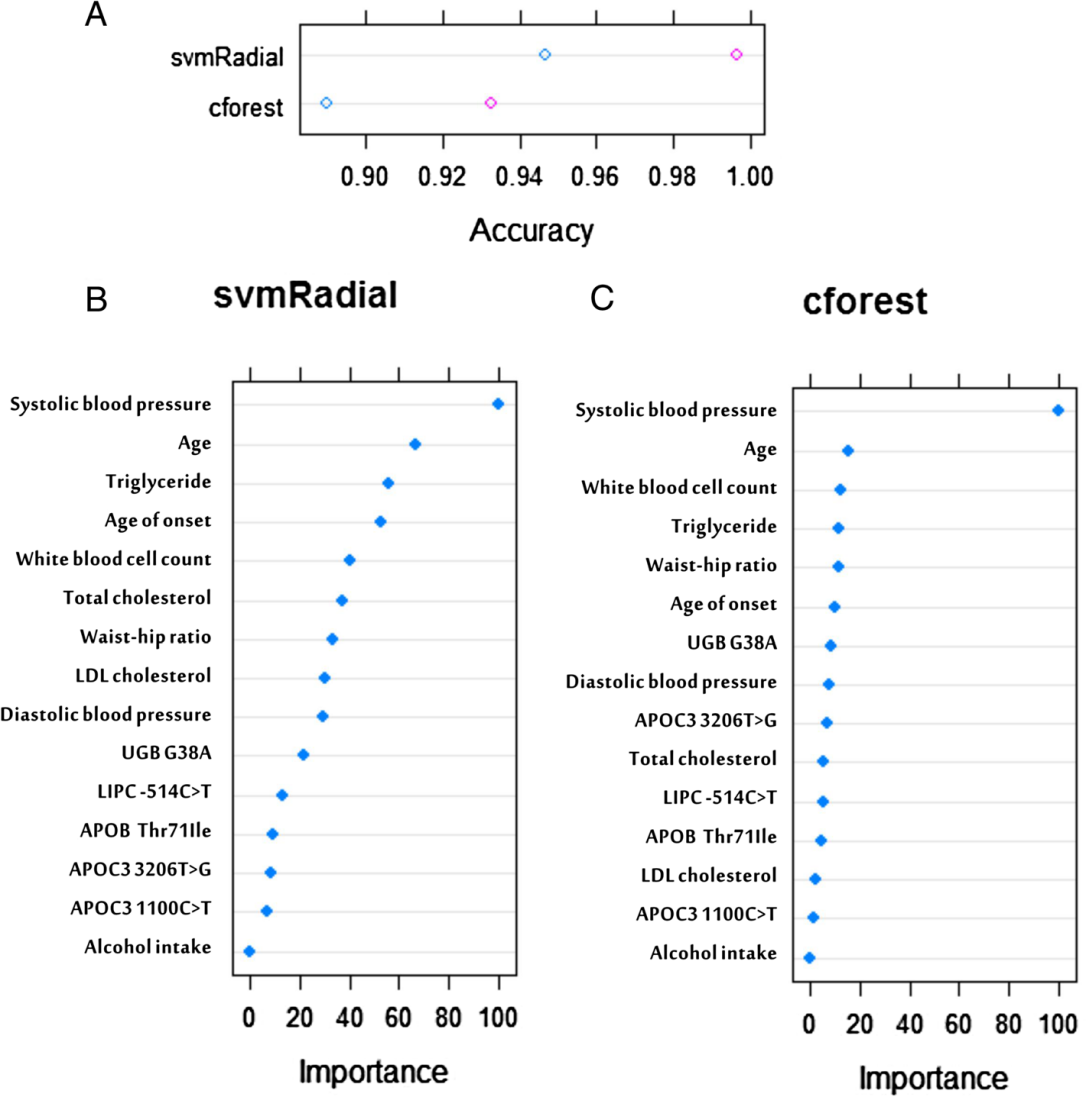
**Prediction performance by support vector machine (svmRadial) and random forest (cforest) using 10 and 5 most frequently selected clinical and genetic attributes respectively. A)** prediction accuracy in the DKD training and testing datasets, **B)** ranking of importance of attributes based on svmRadial, **C)** ranking of importance of attributes based on cforest.

Using this strategy, we identified 10 clinical attributes (systolic BP, age, age of diagnosis, triglyceride [TG], white blood cell count [WBC], TC, waist to hip ratio [WHR], LDL cholesterol [LDL], diastolic BP and alcohol intake) and 5 genetic attributes (*UGB G38A*, *LIPC* -*514C* > *T*, *APOB Thr71Ile*, *APOC3 3206T* > *G* and *APOC3 1100C* > *T*) to build the final models (Figure 3). With this smaller number of attributes, there was a slight drop in accurarcy for the cforest (Figure 3A), while svmRadial maintained its prediction accuracy.

## Discussion

In this 8-year prospective cohort of 1386 type 2 diabetic patients, after excluding well known parameters predictive of DKD including urinary ACR, serum creatinine and drug use, we selected 673 patients with 17 clinical and 70 genetic attributes and performed extensive analyses using seven popular machine learning methods. Age, age of diagnosis, systolic BP and polymorphisms of genes implicated in inflammation and lipid metabolism were most frequently selected by all machine learning methods. Using 10-fold cross validation for parameter optimization and resampling analysis for evaluation, support vector machine and random forest outperformed the other machine learning methods. Using the best predictors from these two models, we were able to select 10 clinical and 5 genetic attributes to predict DKD.

One objective of the study was to compare the performance of different machine learning methods. We used two tree-based models (recursive partitioning/regression trees and C5.0 decision tree) to explore the structure of the dataset which only showed average performance. Random forest performed better than the two tree methods, but required long computation time probably due to the generation of a large number of bootstrapped trees for decision making. Naïve Bayes classifier had the largest variations in performance even during the training stage, indicating that this method was highly sensitive to data input. Neural network had excellent prediction accuracy comparable to those of support vector machine and random forest, but this was not maintained when only clinical attributes were included. Support vector machine was the best performing machine learning method using clinical, genetic or both attributes. The contrast between the performance of neural network and support vector machine using clinical attributes suggested that clinical attributes might generate optimal solutions detected only by a specific method.

With rapid advancement of molecular technology, large datasets containing many genotypes and phenotypes are now available. However, there are major challenges in synthesizing these discoveries and translating them to clinical practice. Thus, our second objective was to determine the best combination of genetic and clinical attributes to predict DKD. Indeed, all machine learning methods preferentially selected clinical, notably, age, age of diagnosis and systolic BP, over genetic attributes for model building. These findings suggested at least in subjects with phenotypes predictive of DKD, the predictive value of genetic factors might be attenuated. These findings also reinforced our current understanding that apart from age, early diagnosis and optimal control of BP are the most effective preventive measures to reduce risk of DKD.

The third objective of this analysis was to develop a novel method to increase the utility of these predictors. Using the top 10 clinical attributes and 5 genetic attributes selected by the best models, we were able to build models using support vector machine and random forest to generate high-performance models. In keeping with our previous reports regarding the importance of metabolic syndrome [1] characterized by central obesity, dyslipidemia and inflammation [18-20] in predicting DKD using conventional correlation and regression analyses, the most predictive clinical factors were lipids, WHR and WBC count in our final models. The co-selection of *LIPC* -*514C* > *T*, *APOB Thr71Ile* and *UGB G38A* polymorphisms were also in accord with our previous reports using regression analysis [21,22]. Our current study highlighted that optimization method such as genetic algorithm could be used to explore genotype-phenotype interactions using a smaller set of attributes for DKD.

Of note, the steroid-inducible protein, *UGB*, have known immunomodulatory and regulatory roles in the deposition of fibronectin and collagen in mouse glomeruli. Herein, DKD is characterized by glomerulopathy with glomerular sclerosis, thickening of basement membrane and mesangial expansion. In *UGB* knockout mice, the animal developed severe renal disease due to abnormal deposition of fibronectin and collagen in the glomeruli [23]. In a Japanese study, association of *UGB G38A* polymorphism with progression of IgA nephropathy has been reported [24]. Taken together, our results support the increasing recognition regarding the pathogenetic role of metabolic syndrome characterized by lipotoxicity and inflammation in DKD [25,26].

The strengths of our study included the extensive phenotypes and genotypes and definition of DKD using prospective design. However, our study has several limitations. Firstly, we did not include eGFR and urinary ACR in order to discover novel predictors. Secondly, we excluded drug treatment which was selected by all machine learning methods, likely confounded by drug indications. Thirdly, this was a proof-of-concept study and the results from this exploratory analysis required independent replication in larger cohorts. Lastly, all participants were of Chinese ethnicity and thus the results might not be applicable to non-Chinese population and individuals without diabetes.

## Conclusions

Using a prospective database, we compared the performance of seven common machine learning methods to build models with the optimal combinations of clinical and genetic predictors for DKD. Amongst them, support vector machine and random forest had the best performance. Age, age of diagnosis and lipid parameters were major clinical predictors while genetic polymorphisms related to inflammation and lipid metabolism were the most selected genetic predictors. Validation of these genetic markers in subjects without clinical evidence of renal disease may provide an opportunity to identify high risk subjects for regular surveillance and individualized treatment including control of inflammation [27] and dyslipidemia [28] to prevent DKD.

## Abbreviations

ACEI: Angiotensin-converting enzyme inhibitor; ACR: Albumin to creatinine ratio; ARB: Angiotensin receptor blockers; AUC: Area under the curve; BP: Blood pressure; cforest: Random forest; DKD: Diabetic kidney disease; eGFR: Estimated glomerular filtration rate; GWAS: Genome wide association studies; IQR: Inter-quartile range; LDL: LDL cholesterol; NB: Naïve bayes classification; NNET: Neural network; PLS: Partial least squares regression; ROC: Receiver operator curve; SNP: Single nucleotide polymorphism; svmRadial: Support vector machine; TC: Total cholesterol; TG: Triglyceride; WBC: White blood cell count; WHR: Waist to hip ratio.

## Competing interests

The authors declared that they had no competing interests.

## Authors’ contributions

JCNC, SKWT, RCWM, WYS and MCYN have participated in the study design, acquisition of data and interpretation of data. YW, VL and MCYN have performed the SNPs genotype. RKKL, YW, and AOYL have participated in data analysis and interpretation of data. RKKL, YW, AOYL and JCNC have participated in paper writing and revision. JCNC and SKWT gave final approval of the version to be published. All authors have read and approved the final manuscript.

## Authors’ information

^1^Hong Kong Bioinformatics Centre, ^2^Department of Medicine and Therapeutics, ^3^Li Ka Shing Institute of Health, ^4^Hong Kong Institute of Diabetes and Obesity, The Chinese University of Hong Kong.

## Pre-publication history

The pre-publication history for this paper can be accessed here:

http://www.biomedcentral.com/1471-2369/14/162/prepub

## Supplementary Material

Additional file 1 Minor allele frequencies of 79 SNPs of 55 candidate genes related to cardiovascular disease and inflammation analysed in 1386 Chinese patients with type 2 diabetes. Click here for file

Additional file 2 Package versions and parameters of machine learning methods used in the present study. Click here for file

Additional file 3 Comparison of baseline clinical and biochemical characteristics of type 2 diabetic patients included in the machine learning analysis (N = 673) and the excluded patients (N = 713) due to incomplete dataset. Click here for file

Additional file 4 **Ranking of importance of variables by different machine learning models (information unavailable in nnet and nb), using A) clinical and genetic attributes, B) genetic-only attributes, C) clinical-only attributes.** Click here for file
