# Supplementary material for: Using a multi-staged strategy based on machine learning and mathematical modeling to predict genotype-phenotype risk patterns in diabetic kidney disease: a prospective case–control cohort analysis
Source: BMC Nephrol. 2013 Jul 23;14:162. doi: 10.1186/1471-2369-14-162 (PMC3726338; doi:10.1186/1471-2369-14-162)
Supplement: Additional file 1 — Minor allele frequencies of 79 SNPs of 55 candidate genes related to cardiovascular disease and inflammation analysed in 1386 Chinese patients with type 2 diabetes. [file 1471-2369-14-162-S1.doc]

**Additional file 1.** Minor allele frequencies of 79 SNPs of 55 candidate genes related to cardiovascular disease and inflammation analysed in 1386 Chinese patients with type 2 diabetes

| **Gene symbol** | **Gene name** | **Polymorphism** | | **Minor allele frequency** | **rs#** |
| --- | --- | --- | --- | --- | --- |
| ***Inflammation*** | | | | | |
| *IL4* | Interleukin 4 | | *582C>T* | 0.200 | rs2243250 |
| *IL4R* | Interleukin 4 receptor | | *398A>G* | 0.481 | rs1805010 |
|  |  | | *1682T>C* | 0.075 | rs1805015 |
|  |  | | *1902A>G* | 0.171 | rs1801275 |
| *IL13* | Interleukin 13 | | *4045C>T* | 0.336 | rs1295686 |
| *UGB* | Uteroglobin | | *G38A* | 0.365 | rs3741240 |
| *SCYA11* | Eotaxin | | *1169G>A* | 0.076 | rs4795895 |
|  |  | | *Ala23Thr* | 0.192 | rs3744508 |
| *CCR2* | Monocyte chemotactic protein 1 receptor | | *46295G>A* | 0.183 | rs1799864 |
| *CCR5* | CC chemokine receptor 5 | | *59029G>A* | 0.359 | rs1799987 |
| *TCF7* | Transcription factor 7 | | *383A>T* | 0.176 | rs244656 |
| *IL1A* | Interleukin 1α | | *549T>C* | 0.067 | rs1800587 |
| *IL1B* | Interleukin 1β | | *1423C>T* | 0.488 | rs16944 |
|  |  | | *4336C>T* | 0.011 | rs1143634 |
| *IL5RA* | Interleukin 5 receptor alpha | | *-5091G>A* | 0.189 | rs2290608 |
| *IL6* | Interleukin 6 | | *589G>C* | 0.237 | rs1800796 |
| *IL10* | Interleukin 10 | | *-597C>A* | 0.296 | rs1800872 |
| *SDF1* | Stromal cell-derived factor 1 | | *880G>A* | 0.280 | rs1801157 |
| *CD14* | CD14 | | *2232C>T* | 0.420 | rs2569190 |
| *C5* | Complement component 5 | | *2416A>G* | 0.425 | rs17611 |
| *CSF2* | Colony-stimulating factor 2 | | *2600T>C* | 0.388 | rs25882 |
| *CTLA4* | Cytotoxic T lymphocyte-associated 4 | | *875C>T* | 0.110 | rs5742909 |
|  |  | | *Thr17Ala* | 0.354 | rs231775 |
| *LTC4S* | Leukotriene C4 synthase | | *620A>C* | 0.158 | rs730012 |
| *VDR* | Vitamin D receptor | | *12022T>C* | 0.468 | rs2228570 |
|  |  | | *45082G>A* | 0.050 | rs1544410 |
| *GC* | Group-specific component | | *Glu432Asp* | 0.307 | rs7041 |
|  |  | | *Thr436Lys* | 0.270 | rs4588 |
| ***Lipid metabolism*** | | | | | |
| *APOB* | Apolipoprotein B | *Thr71Ile* | | 0.147 | rs1367117 |
| *APOC3* | Apolipoprotein CIII | *-641C>A* | | 0.440 | rs2542052 |
|  |  | *-482C>T* | | 0.425 | rs2854117 |
|  |  | *-455T>C* | | 0.424 | rs2854116 |
|  |  | *1100C>T* | | 0.396 | rs4520 |
|  |  | *3175C>G* | | 0.314 | rs5128 |
|  |  | *3206T>G* | | 0.229 | rs4225 |
| *APOE* | Apolipoprotein E e4/e3/e2 | *Cys112Arg* | | 0.084 | rs429358 |
|  |  | *Arg158Cys* | | 0.094 | rs7412 |
| *LIPC* | Hepatic lipase | *-514C>T* | | 0.375 | rs1800588 |
| *LPL* | Lipoprotein lipase | *Ser447Ter* | | 0.122 | rs328 |
| *PON1* | Paraoxonase 1 | *Met55Leu* | | 0.475 | rs3202100 |
|  |  | *Gln192Arg* | | 0.350 | rs662 |
| *PON2* | Paraoxonase 2 | *Ser311Cys* | | 0.196 | rs7493 |
| *LDLR* | LDL receptor | Exon 18 *NcoI* | | 0.373 | rs5742911 |
| *CETP* | Cholesteryl ester transfer protein | *-628C>A* | | 0.461 | rs1800775 |
|  |  | *Ile405Val* | | 0.430 | rs5882 |
|  |  | *Asp442Gly* | | 0.022 | rs2303790 |
| *PPARG* | Peroxisome proliferator activated receptor γ | *Pro12Ala* | | 0.028 | rs1801282 |
| ***Thrombosis/Homocysteine*** | | | | | |
| *F7* | Factor VII | (-323)10bp *del/ins* | | 0.037 | rs5742910 |
|  |  | *Arg353Gln* | | 0.036 | rs6046 |
| *PAI-1* | Plasminogen activator inhibitor-1 | *(-675) 5G/4G* | | 0.446 | rs1799768 |
|  |  | *11053G>T* | | 0.449 | rs7242 |
| *FGB* | Fibrinogen | *β chain -455G>A* | | 0.277 | rs1800790 |
| *ITGA2* | Glycoprotein Ia | *873G>A* | | 0.288 | rs1062535 |
| *GNB3* | G protein β3 subunit | *825C>T* | | 0.447 | rs5443 |
| *MTHFR* | 5,10-Methylenetetrahydrofolate reductase | *Ala222Val* | | 0.229 | rs1801133 |
| ***Endothelial function/Cytokine*** | | | | | |
| *ICAM1* | Intracellular adhesion molecule 1 | *Lys56Met* | | 0.050 | rs5491 |
| *VCAM1* | Vascular cell adhesion molecule 1 | *707T>C* | | 0.158 | rs1041163 |
| *SELE* | Endothelial adhesion molecule 1 | *Ser128Arg* | | 0.010 | rs5361 |
|  |  | *Leu554Phe* | | 0.039 | rs5355 |
| *SELP* | Selectin P | *Ser331Asn* | | 0.231 | rs6131 |
| *FCERB1* | Immunoglobulin E receptor 1β | *Glu237Gly* | | 0.202 | rs569108 |
| *TGFB1* | Transforming growth factor β1 | *629C>T* | | 0.429 | rs1800469 |
| *NOS2* | Nitric oxide synthase 2A | *231C>T* | | 0.194 | rs1137933 |
| *NOS3* | Endothelial nitric oxide synthase | *-922A>G* | | 0.123 | rs1800779 |
|  |  | *Glu298Asp* | | 0.114 | rs1799983 |
| *LTA* | Lymphotoxin α | *1069A>G* | | 0.449 | rs909253 |
|  |  | *Thr26Asn* | | 0.448 | rs1041981 |
| *TNF* | Tumor necrosis factor α | *-308G>A* | | 0.089 | rs1800629 |
|  |  | *-244G>A* | | 0.024 | rs673 |
| ***Stress & Natricuretic hormone*** | | | | | |
| *AGTR1* | Angiotensin II receptor, type 1 | *1166A>C* | | 0.047 | rs5186 |
| *AGT* | Angiotensinogen | *Met235Thr* | | 0.153 | rs699 |
| *ADRB2* | β2-adrenergic receptor | *Arg16Gly* | | 0.412 | rs1042713 |
| *NPPA* | Atrial natriuretic peptide | *644G>A* | | 0.081 | rs1042714 |
| *ADRB3* | β3-adrenergic receptor | *Trp64Arg* | | 0.124 | rs4994 |
| *NPPA* | Atrial natriuretic peptide | *644G>A* | | 0.129 | rs5063 |
| *ADD1* | α-adducin | *Gly460Trp* | | 0.411 | rs4961 |
| *SCNN1A* | Epithelial sodium channel α subunit | *Thr663Ala* | | 0.455 | rs2228576 |
| ***Polyol pathway*** | | | | | |
| *ALR2* | Aldose reductase | Promoter *(CA)n* | | - | - |
|  |  | *-106C>T* | | 0.220 | rs759853 |
