# Supplementary material for: Using a multi-staged strategy based on machine learning and mathematical modeling to predict genotype-phenotype risk patterns in diabetic kidney disease: a prospective case–control cohort analysis
Source: BMC Nephrol. 2013 Jul 23;14:162. doi: 10.1186/1471-2369-14-162 (PMC3726338; doi:10.1186/1471-2369-14-162)
Supplement: Additional file 2 — Package versions and parameters of machine learning methods used in the present study. [file 1471-2369-14-162-S2.doc]

**Additional file 2.** Package versions and parameters of machine learning methods used in the present study

The details of package versions (in brackets) and parameters used for each machine learning method were described as follows. The ones with asterisk were tested in cross-validation. C5.0 decision tree (0.1.0-13): subset = TRUE, bands = 0, winnow = FALSE, noGlobalPruning = FALSE, CF = 0.25, minCases = 2, fuzzyThreshold = FALSE, sample = 0, earlyStopping = TRUE. naïve Bayes classification (e1071:1.6-1): Laplace smoothing = FALSE, Value replacing cells with 0 probabilities = 0.001*, and kernel density estimate for density estimation = TRUE*. Feed-forward neural networks (7.3-5): model = multinomial log-linear models: maximum number of iterations = 100, fitting = least squares, Initial random weights = 0.7, The maximum allowable number of weights = 1000, absolute stop fit criterion = 1.0^e-4^, relative stop fit criterion = 1.0^e-8^, size of single hidden layers = 11* and weight decay = 0.1*. The classification and regression tree: minsplit = 20, minbucket = round(minsplit/3), maxcompete = 4, maxsurrogate = 5, usesurrogate = 2, xval = 10, surrogatestyle = 0, maxdepth = 30 and cp = 0.00939*. Partial least square regression (2.3-0): The fit method to use in partial least square regression = kernalpls, The tolerance used for removing values close to 0 in the vectors of loading weights = the smallest positive floating-point number, The tolerance used for removing predictor variables with L1 norms close to 0 =10^-12 and ncomp = 9*. Random forest (cforest 1.0-6): teststat = "max", testtype = "Teststatistic", mincriterion = qnorm(0.9), savesplitstats = FALSE, ntree = 500, replace = TRUE, fraction = 0.632, trace = FALSE, mtry = 5*. Support vector machine (kernlab 0.9-15): kernel = Gaussian radial basis function, C = 4* and sigma = 0.0112*.
