# Supplementary material for: Using a multi-staged strategy based on machine learning and mathematical modeling to predict genotype-phenotype risk patterns in diabetic kidney disease: a prospective case–control cohort analysis
Source: BMC Nephrol. 2013 Jul 23;14:162. doi: 10.1186/1471-2369-14-162 (PMC3726338; doi:10.1186/1471-2369-14-162)
Supplement: Additional file 3 — Comparison of baseline clinical and biochemical characteristics of type 2 diabetic patients included in the machine learning analysis (N = 673) and the excluded patients (N = 713) due to incomplete dataset. [file 1471-2369-14-162-S3.doc]

**Additional file 3.** Comparison of baseline clinical and biochemical characteristics of type 2 diabetic patients included in the machine learning analysis (N=673) and the excluded patients (N=713) due to incomplete dataset.

|  | **Subjects included in the analysis** | **Subjects excluded from the analysis** | **P value** |
| --- | --- | --- | --- |
| Number | 673 | 713 |  |
| **Clinical features** |  |  |  |
| Age (years) | 57  (48 to 65) | 60  (48 to 67) | 0.004 ^b^ |
| Male sex | 58.8%(396) | 56.2%(401) | 0.328 ^a^ |
| Age of onset (years) | 46  (39 to 56) | 50  (40 to 60) | <0.001 ^b^ |
| Duration of diabetes (years) | 9  (3 to 13) | 7  (2 to 12) | <0.001^b^ |
| Smoking |  |  | 0.173 ^a^ |
| Ex smokers | 29.7%(200) | 31.6%(225) |  |
| Current smokers | 11.6%(78) | 14.2%(101) |  |
| BMI (kg/m^2^) | 24.8  (22.4 to 27.1) | 24.2  (22.2 to 26.4) | 0.046 ^b^ |
| Waist circumference (cm) Men | 88.0  (83.0 to 93.0) | 87.0  (81.0 to 93.0) | 0.380 ^b^ |
| Waist circumference (cm) Women | 83.0  (77.0 to 89.0) | 82.0  (76.0 to 89.0) | 0.060 ^b^ |
| Waist to hip ratio | 0.89  (0.84 to 0.93) | 0.89  (0.84 to 0.93) | 0.781 ^b^ |
| Systolic BP (mmHg) | 136  (122 to 151) | 135  (122 to 155) | 0.391 ^b^ |
| Diastolic BP (mmHg) | 79  (70 to 86) | 79  (70 to 86) | 0.788 ^b^ |
| **Laboratory data** |  |  |  |
| HbA1c (%) | 7.6  (6.7 to 8.8) | 7.5  (6.6 to 9.0) | 0.786 ^b^ |
| Fasting plasma glucose (mmol/l) | 7.9  (6.4 to 10.4) | 8.2  (6.5 to 10.7) | 0.260 ^b^ |
| LDL cholesterol (mmol/l) | 3.30  (2.70 to 4.00) | 3.40  (2.90 to 4.20) | 0.021 ^b^ |
| HDL cholesterol (mmol/l) | 1.20  (1.00 to 1.50) | 1.20  (1.00 to 1.50) | 0.447 ^b^ |
| Triglyceride (mmol/l) | 1.33  (0.91 to 2.07) | 1.43  (0.94 to 2.12) | 0.237 ^b^ |
| Total cholesterol (mmol/l) | 5.3  (4.7 to 6.1) | 5.4  (4.7 to 6.3) | 0.028 ^b^ |
| White blood cell count (×10^9^/L) | 7.2  (6.0 to 8.5) | 7.1  (6.1 to 8.4) | 0.901 ^b^ |
| ACR (mg/mmol) | 2.0  (0.9 to 39.0) | 4.1  (0.9 to 82.9) | <0.001 ^b^ |
| eGFR (ml/min per 1.73m^2^) | 111.4  (88.0 to 132.8) | 103.4  (73.5 to126.9) | <0.001 ^b^ |
| **Drug use at baseline** |  |  |  |
| Lipid lowering drugs | 8.6%(58) | 5.6%(40) | 0.029 ^a^ |
| ACEI/ARB | 10.1%(68) | 10.5%(75) | 0.800 ^a^ |
| Other blood pressure lowering drugs | 28.8%(194) | 25.8%(184) | 0.207 ^a^ |
| Oral blood glucose lowering drugs | 50.2%(338) | 47.4%(340) | 0.345^a^ |
| Insulin | 20.8%(140) | 17.1%(100) | 0.079^a^ |
| **Clinical outcomes** |  |  |  |
| Incident renal endpoint | 8.5%(47) | 30.9%(188) | <0.001^a^ |
| Incident cardiovascular endpoint | 15.4%(89) | 12.4%(73) | 0.147 ^a^ |

^a^, Derived from Chi-square text, %(N); ^b^, Mann-Whitney Two-Sample Test, Median (25^th^ to 75^th^ quartiles).

Abbreviation: BMI, body mass index; HbA1c, glycated hemoglobin; HDL, high density lipoprotein; LDL, low density lipoprotein.
