# Supplementary material for: Using a multi-staged strategy based on machine learning and mathematical modeling to predict genotype-phenotype risk patterns in diabetic kidney disease: a prospective case–control cohort analysis
Source: BMC Nephrol. 2013 Jul 23;14:162. doi: 10.1186/1471-2369-14-162 (PMC3726338; doi:10.1186/1471-2369-14-162)

**Additional file 4.** Variable importance based on different machine learning models (information unavailable in nnet and nb), using A) clinical and genetic attributes, B) genetic-only attributes, C) clinical-only attributes

Abbreviations – AGE_ONES: Age of onset; ALCOH_RC: Alcohol intake; SMOK_RC: smoking status; DMAGE: duration of diabetes; UGBi: *UGB G38A;* LIPCc: *LIPC -514C>T;* APOB1c: *APOB Thr71Ile;* APOC36c: *APOC3 3206T>G;* APOC34c: *APOC3 1100C>T;* IL1B1i: *IL1B 1423C>T;* CTLA41i: *CTLA4 Thr17Ala;* IL5RAi: *IL5RA -5091G>A;* IL4R1i: *IL4R 398A>G;* VDR1i: *VDR 12022T>C;* IL10i: *IL10 -597C>A;* GNB3c: *GNB3825C>T;* LTC4Si: *LTC4S 620A>C;* SCY11i: *SCYA11 1169G>A;* NOS2Ai: *NOS2 231C>T;* SELP1i: *SELP Ser331Asn;* SCNN1A2c: *SCNN1A Thr663Ala;* CTLA41i: *CTLA4 875C>T;* GC_H: *GC Glu432Asp & Thr436Lys;* CD14i: *CD14 2232C>T;* ALR_CT: *ALR2 -106C>T;* IL1Ai: *IL1A 549T>C;* APOEc_H: *APOE e4/e3/e2;* NPPA1c: *NPPA 644G>A;* ADD1c: *ADD1 Gly460Trp;* PON12c: *PON1 Gln192Arg;* NOS31f: *NOS3 -922A>G;* ITGA2c: *ITGA2 873G>A;* IL13i: *IL134045C>T;* APOC32c: *APOC3 -482C>T;* CETP2c: *CETP Ile405Val;* FCER1Bi: *FCERB1 Glu237Gly;* LPL4c: *LPL Ser447Ter;* SDF1i: *SDF1 880G>A;* CETP1c_H: *CETP Ile405Val & Asp442Gly;* CCR2i: *CCR2 46295G>A;* VDR2i: *VDR 45082G>A.* UGBi: *UGB G38A;* LIPCc: *LIPC -514C>T;* APOB1c: *APOB Thr71Ile;* APOC36c: *APOC3 3206T>G;* APOC34c: *APOC3 1100C>T;* IL1B1i: *IL1B 1423C>T;* CTLA42i: *CTLA4 Thr17Ala;* IL5RAi: *IL5RA -5091G>A;* IL4R1i: *IL4R 398A>G;* VDR1i: *VDR 12022T>C;* IL10i: *IL10 -597C>A;* GNB3c: *GNB3825C>T;* LTC4Si: *LTC4S 620A>C;* SCY11i: *SCYA11 1169G>A;* NOS2Ai: *NOS2 231C>T;* SELP1i: *SELP Ser331Asn;* SCNN1A2c: *SCNN1A Thr663Ala;* CTLA42i: *CTLA4 875C>T;* GC_H: *GC Glu432Asp & Thr436Lys;* CD14i: *CD14 2232C>T;* ALR_CT: *ALR2 -106C>T;* IL1Ai: *IL1A 549T>C;* APOEc_H: *APOE e4/e3/e2;* NPPA1c: *NPPA 644G>A;* ADD1c: *ADD1 Gly460Trp;* PON12c: *PON1 Gln192Arg;* NOS31f: *NOS3 -922A>G;* ITGA2c: *ITGA2 873G>A;* IL13i: *IL134045C>T;* APOC32c: *APOC3 -482C>T;* CETP2c: *CETP Ile405Val;* FCER1Bi: *FCERB1 Glu237Gly;* LPL4c: *LPL Ser447Ter;* SDF1i: *SDF1 880G>A;* CETP1c_H: *CETP Ile405Val & Asp442Gly;* CCR2i: *CCR2 46295G>A;* VDR2i: *VDR 45082G>A;* APOC32c: *APOC3-482C>T;* CETP2c: *CETP Ile405Val;* CA_Genotype: *ALR2(CA)n;* PON2c: *PON2 Ser311Cys;* SELE2c: *SELE Leu554Phe;* NOS33f: *NOS3 Glu298Asp;* IL4R3i: *IL4R 1902A>G;* CCR52i: *CCR5 59029G>A;* TGFB1i: *TGFB1 629C>T;* LTAf: *LTA Thr26Asn;* PAI12c: *PAI-1 11053G>T;* ADRB22C: *ADRB2 Gln27Glu.*

A)
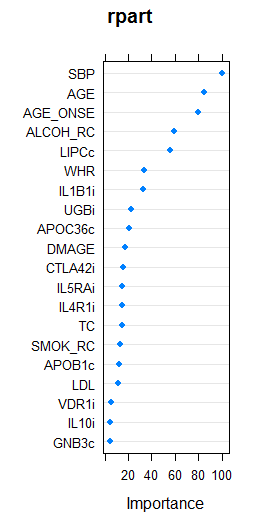

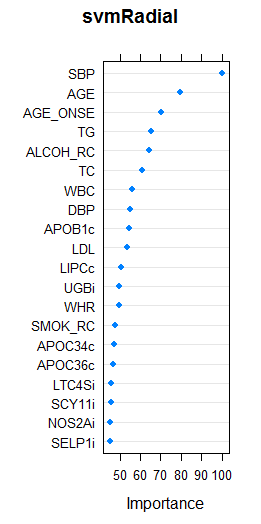

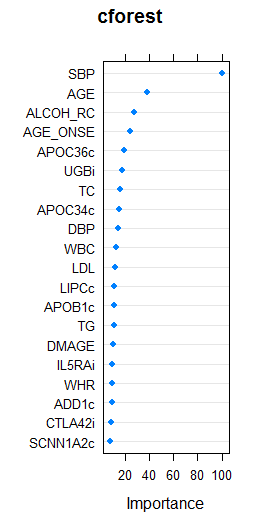

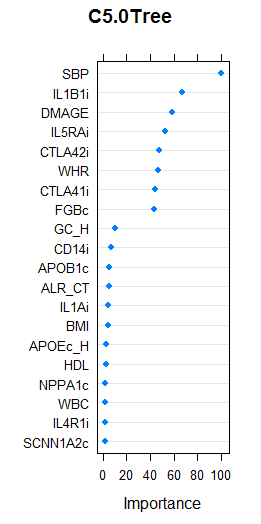

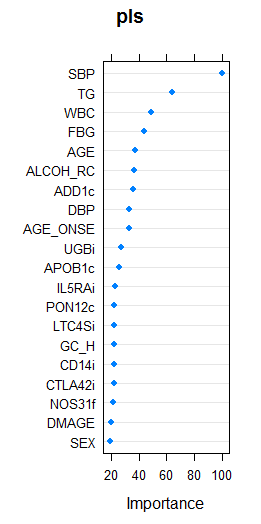


B)
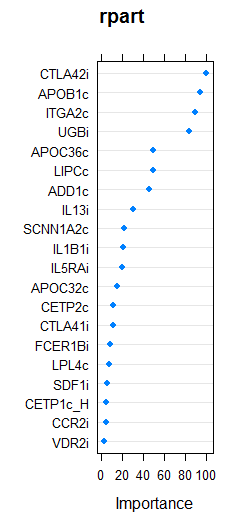

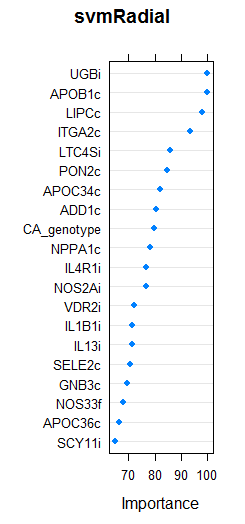

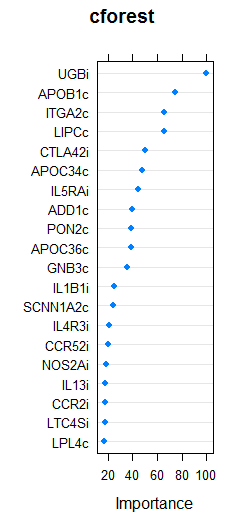

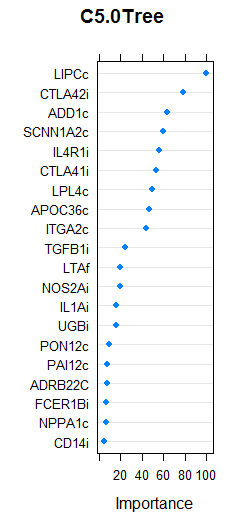

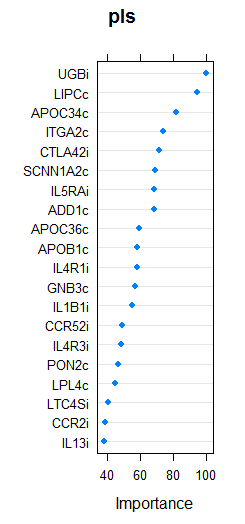


C)
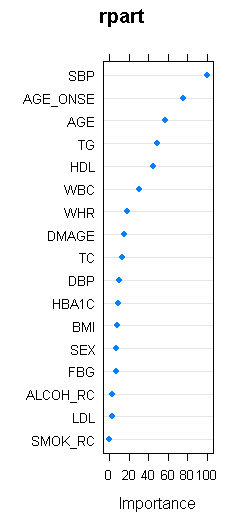

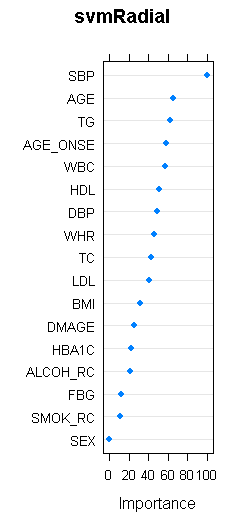

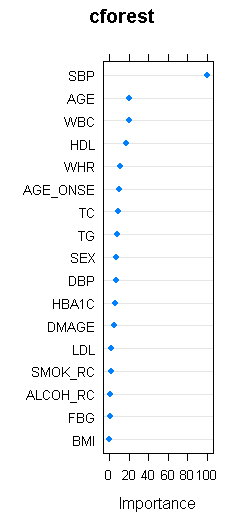

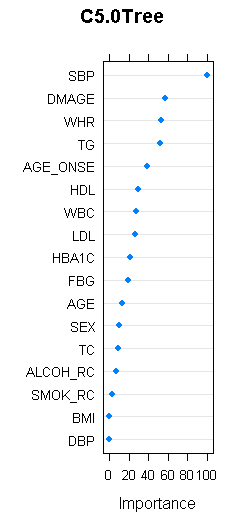

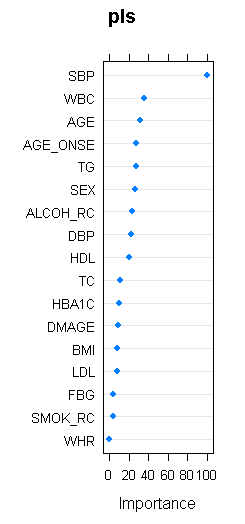

Supplement: Additional file 4 — Ranking of importance of variables by different machine learning models (information unavailable in nnet and nb), using A) clinical and genetic attributes, B) genetic-only attributes, C) clinical-only attributes. [file 1471-2369-14-162-S4.doc]
